# Transmission eigenvalue distributions in highly conductive molecular junctions

**DOI:** 10.3762/bjnano.3.5

**Published:** 2012-01-16

**Authors:** Justin P Bergfield, Joshua D Barr, Charles A Stafford

**Affiliations:** 1 Departments of Chemistry and Physics, University of California, Irvine, California 92697, USA; 2 Department of Physics, University of Arizona, 1118 East Fourth Street, Tucson, AZ 85721, USA

**Keywords:** benzene–platinum junction, effective-field theory, isolated-resonance approximation, lead–molecule interface, many-body theory, multichannel, quantum transport, single-molecule junction, transmission eigenchannels

## Abstract

**Background:** The transport through a quantum-scale device may be uniquely characterized by its transmission eigenvalues τ*_n_*. Recently, highly conductive single-molecule junctions (SMJ) with multiple transport channels (i.e., several τ*_n_* > 0) have been formed from benzene molecules between Pt electrodes. Transport through these multichannel SMJs is a probe of both the bonding properties at the lead–molecule interface and of the molecular symmetry.

**Results:** We use a many-body theory that properly describes the complementary wave–particle nature of the electron to investigate transport in an ensemble of Pt–benzene–Pt junctions. We utilize an effective-field theory of interacting π-electrons to accurately model the electrostatic influence of the leads, and we develop an ab initio tunneling model to describe the details of the lead–molecule bonding over an ensemble of junction geometries. We also develop a simple decomposition of transmission eigenchannels into molecular resonances based on the isolated resonance approximation, which helps to illustrate the workings of our many-body theory, and facilitates unambiguous interpretation of transmission spectra.

**Conclusion:** We confirm that Pt–benzene–Pt junctions have two dominant transmission channels, with only a small contribution from a third channel with τ*_n_* << 1. In addition, we demonstrate that the isolated resonance approximation is extremely accurate and determine that transport occurs predominantly via the HOMO orbital in Pt–benzene–Pt junctions. Finally, we show that the transport occurs in a lead–molecule coupling regime where the charge carriers are both particle-like and wave-like simultaneously, requiring a many-body description.

## Introduction

The number of transmission channels for a single-atom contact between two metallic electrodes is simply given by the chemical valence of the atom [1]. Recently, it was argued [2] that the number of dominant transmission channels in a single-molecule junction (SMJ) is determined by the degeneracy of the molecular orbital [3] closest to the metal Fermi level. In this article, we focus on ensembles of highly conductive Pt–benzene–Pt junctions [4] in which the lead and molecule are in direct contact. Going beyond the phenomenological random-matrix model of lead–molecule coupling considered in [2], a realistic atomistic model is developed to describe lead–molecule coupling over an ensemble of energetically favored junction geometries.

For a two-terminal SMJ, the transmission eigenvalues τ*_n_* are eigenvalues of the elastic transmission matrix [5]

[1]
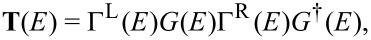


where *G* is the retarded Green’s function [6] of the SMJ, Γ*^α^* is the tunneling-width matrix describing the bonding of the molecule to lead *α*, and the total transmission function *T*(*E*) = Tr{**T**(*E*)}. The number of transmission channels is equal to the rank of the matrix (Equation 1), which is in turn limited by the ranks of the matrices *G* and Γ*^α^* [2]. The additional two-fold spin degeneracy of each resonance is considered to be implicit throughout this work. As indicated by Equation 1, an accurate description of transport requires an accurate result for *G*, which can be calculated by using either single-particle or many-body methods, and which depends critically on accurate descriptions of the molecular energy levels and the lead–molecule coupling.

In effective single-particle theories, including current implementations of density functional theory (DFT), it is often necessary [7–10] to describe the transport problem by considering an “extended molecule”, composed of the molecule and several electrode atoms. This procedure makes it difficult, if not impossible, to assign transmission eigenchannels to individual molecular resonances since the quantum states of the extended molecule bear little resemblance to the states of the molecule itself.

We utilize a nonequilibrium many-body theory based on the molecular Dyson equation (MDE) [6] to investigate transport distributions of SMJ ensembles. Our MDE theory correctly accounts for wave–particle duality of the charge carriers, simultaneously reproducing the key features of both the Coulomb blockade and coherent-transport regimes, alleviating the necessity of constructing an extended molecule. Consequently, we can unambiguously assign transmission eigenchannels to molecular resonances [2].

Previous applications of our MDE theory [6,11–12] to transport through SMJs utilized a semiempirical Hamiltonian [13] for the π-electrons, which accurately describes the gas-phase spectra of conjugated organic molecules. Although this approach should be adequate to describe molecules weakly coupled to metal electrodes, e.g., by thiol linkages, in junctions where the π-electrons bind directly to the metal electrodes [4], the lead–molecule coupling may be so strong that the molecule itself is significantly altered, necessitating a more fundamental molecular model.

In this work, we utilize an *effective field theory of interacting* π-*electrons* (π-EFT), in which the form of the molecular Hamiltonian is derived from symmetry principles and electromagnetic theory (multipole expansion) [14]. The resulting formalism constitutes a state-of-the-art many-body theory that provides a realistic description of lead–molecule hybridization and van der Waals coupling, as well as the screening of intramolecular interactions by the metal electrodes, all of which are essential for a quantitative description of strongly-coupled SMJs [4].

The bonding between the tip of electrode *α* and the molecule is characterized by the tunneling-width matrix Γ*^α^*, where the rank of Γ*^α^* is equal to the number of covalent bonds formed between the two. For example, in a SMJ where a Au electrode bonds to an organic molecule through a thiol group, only a single bond is formed, and there is only one significant transmission channel [15–16]. In Pt–benzene–Pt junctions, however, each Pt electrode forms multiple bonds to the benzene molecule and multiple transmission channels are observed [4]. In such highly conductive SMJs the lead and molecule are in direct contact and the overlap between the π-electron system of the molecule and *all* of the quasi-atomic wavefunctions of the atomically sharp electrode are relevant. Rather than the random-matrix method used in [2], we develop an atomistic approach to bonding in which the nine relevant orbitals for each Pt electrode are included (one *s*, three *p*, and five *d*), representing the evanescent tunneling modes in free space outside the apex atom of each electrode tip. This atomistic model of lead–molecule coupling allows distributions of transport coefficients to be computed directly over an ensemble of junction geometries, supplanting the phenomenological model of lead–molecule coupling employed in [2].

In the next section, we outline the relevant aspects of our MDE theory and derive transport equations in the isolated-resonance approximation. We then develop our atomistic treatment of lead–molecule coupling, in which the electrostatic influence of the leads is treated by π-EFT and the multiorbital lead–molecule bonding is described using the quasi-atomic orbitals of the electrode tip. Finally, the transport distributions for these ensembles of Pt–benzene–Pt junctions are calculated by using both the full molecular Green’s function and within the isolated-resonance approximation. The efficacy of the isolated resonance approximation is investigated in detail.

### Many-body theory of transport

When macroscopic leads are bonded to a single molecule, a SMJ is formed, transforming the few-body molecular problem into a full many-body problem. The bare molecular states are dressed by interactions with the lead electrons when the SMJ is formed, shifting and broadening them in accordance with the lead–molecule coupling.

Until recently [6] no theory of transport in SMJs was available which properly accounted for the *particle and wave* character of the electron, such that the Coulomb blockade and coherent transport regimes were considered “complementary” [10]. Here, we utilize a many-body MDE theory [6,12] based on nonequilibrium Green’s functions (NEGFs) to investigate transport in multichannel SMJs, which correctly accounts for both aspects of the charge carriers.

In order to calculate transport quantities of interest we must determine the retarded Green’s function *G*(*E*) of the junction, which may be written as

[2]
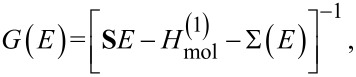


where *H*_mol_ = 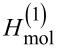 + 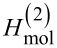 is the molecular Hamiltonian, which we formally separate into one-body and two-body terms [6,12]. **S** is an overlap matrix, which in an orthonormal basis reduces to the identity matrix, and

[3]
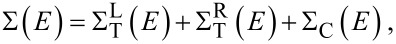


is the self-energy, including the effect of both a finite lead–molecule coupling, through 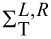, and many-body interactions, through the Coulomb self-energy Σ_C_(*E*). The tunneling self-energy matrices are related to the tunneling-width matrices by

[4]
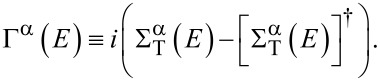


It is useful to define a molecular Green’s function 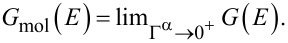 In the sequential tunneling regime [6], where lead–molecule coherences can be neglected, the molecular Green’s function within MDE theory is given by

[5]
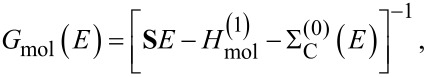


where all one-body terms are included in 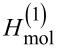 and the Coulomb self-energy Σ^(0)^ accounts for the effect of all intramolecular many-body correlations exactly. The full Green’s function of the SMJ may then be found using the molecular Dyson equation [6]

[6]
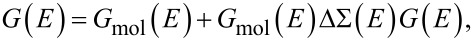


where ΔΣ = Σ_T_ + ΔΣ_C_ and 
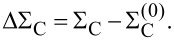
 At room temperature and for small bias voltages, ΔΣ_C_ ≈ 0 in the cotunneling regime [6] (i.e., for nonresonant transport). Furthermore, the inelastic transmission probability is negligible compared to the elastic transmission in that limit.

The molecular Green’s function *G*_mol_ is found by exactly diagonalizing the molecular Hamiltonian, including all charge states and excited states of the molecule [6,12]

[7]
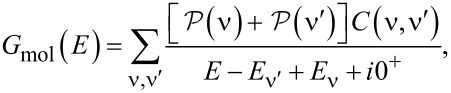


where 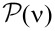 is the probability that the molecular state ν is occupied, *C*(ν,ν′) are many-body matrix elements and 
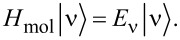
 In linear response, 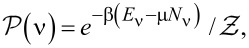 where 
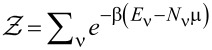
 is the grand canonical partition function.

The rank-1 matrix *C*(ν,ν′) has elements

[8]
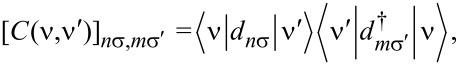


where *d_n_*_σ_ annihilates an electron of spin σ on the *n*th atomic orbital of the molecule, and ν and ν′ label molecular eigenstates with different charge. The rank of *C*(ν,ν′) in conjunction with Equation 6 and Equation 7 implies that each molecular resonance ν → ν′ contributes at most one transmission channel in Equation 1, suggesting that an *M*-fold-degenerate molecular resonance could sustain a maximum of *M* transmission channels.

### Isolated-resonance approximation

Owing to the position of the chemical potential of the leads relative to the molecular energy levels and the large charging energy of small molecules, transport in SMJs is typically dominated by individual molecular resonances. In this subsection, we calculate the Green’s function in the isolated-resonance approximation wherein only a single (nondegenerate or degenerate) molecular resonance is considered. In addition to developing intuition and gaining insight into the transport mechanisms in a SMJ, we also find (cf. Results and Discussion section) that the isolated-resonance approximation can be used to accurately predict the transport.

#### Nondegenerate molecular resonance

If we consider a single non-degenerate molecular resonance then

[9]
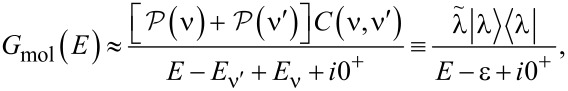


where ε = *E*_ν′_ − *E*_ν_, 
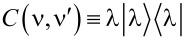
 is the rank-1 many-body overlap matrix, and we have set 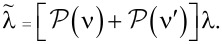 In order to solve *G* analytically, it is useful to rewrite Dyson’s equation (Equation 6) as follows:

[10]
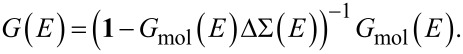


In the elastic-cotunneling regime (ΔΣ_C_ = 0) we find

[11]
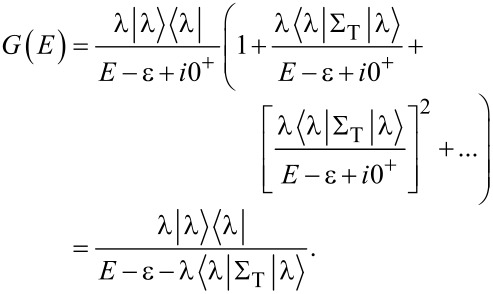


Equation 11 can be equivalently expressed as

[12]
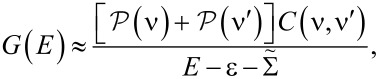


where

[13]
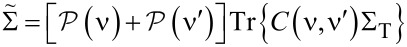


is the effective self-energy at the resonance, which includes the effect of many-body correlations through the *C*(ν,ν′) matrix.

Using Equation 1, the transmission in the isolated-resonance approximation is given by

[14]
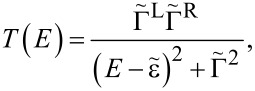


where 
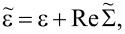


[15]
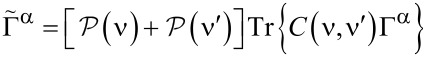


is the dressed tunneling-width matrix, and 
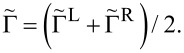


As evidenced by Equation 14, the isolated-resonance approximation gives an intuitive prediction for the transport. Specifically, the transmission function is a single Lorentzian resonance centered about 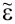 with a half-width at half-maximum of 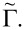 The less-intuitive many-body aspect of the transport problem is encapsulated in the effective tunneling-width matrices 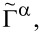 where the overlap of molecular many-body eigenstates can reduce the elements of these matrices and may strongly affect the predicted transport.

#### Degenerate molecular resonance

The generalization of the above results to the case of a degenerate molecular resonance is formally straightforward. For an *M*-fold degenerate molecular resonance

[16]
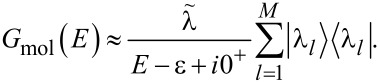


The *M* degenerate eigenvectors of *G*_mol_ may be chosen to diagonalize Σ_T_ on the degenerate subspace

[17]
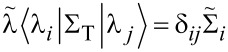


and Dyson’s equation may be solved as before

[18]
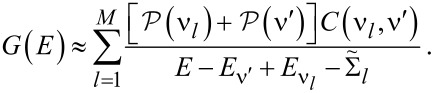


Although Σ_T_ is diagonal in the basis of 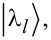 Γ^L^ and Γ^R^ need not be separately diagonal. Consequently, there is no general simple expression for *T*(*E*) for the case of a degenerate resonance, but **T** can still be computed using Equation 1.

In this article we focus on transport through Pt–benzene–Pt SMJs where the relevant molecular resonances (HOMO or LUMO) are doubly degenerate. Considering the HOMO resonance of benzene

[19]
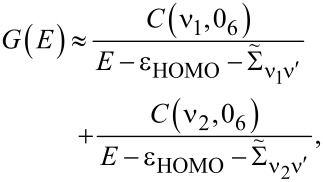


where 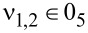 diagonalize Σ_T_ and 0*_N_* is the *N*-particle ground state.

### π-Electron effective field theory

In order to model the degrees of freedom most relevant for transport, we have utilized an effective field theory of interacting π-electron systems (π-EFT) as described in detail in [14]. Briefly, this was done by starting with the full electronic Hamiltonian of a conjugated organic molecule and by dropping degrees of freedom far from the π-electron energy scale. The effective π-orbitals were then assumed to possess azimuthal and inversion symmetry, and the effective Hamiltonian was required to satisfy particle–hole symmetry and be explicitly local. Such an effective field theory is preferable to semiempirical methods for applications in molecular junctions because the effective interaction is derived from Maxwell’s equations, and hence can be readily generalized to include screening of intramolecular Coulomb interactions due to nearby metallic electrodes.

#### Effective Hamiltonian

This allows the effective Hamiltonian for the π-electrons in gas-phase benzene to be expressed as

[20]
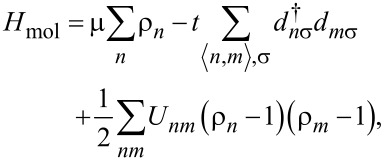


where *t* is the tight-binding matrix element, μ is the molecular chemical potential, *U_nm_* is the Coulomb interaction between the electrons on the *n*th and *m*th π-orbitals, and 
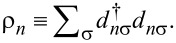
 The interaction matrix *U_nm_* is calculated by way of a multipole expansion keeping terms up to the quadrupole–quadrupole interaction:

[21]
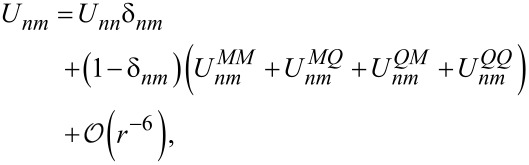


where *U^MM^* is the monopole–monopole interaction, *U^QM^* is the quadrupole–monopole interaction, and *U^QQ^* is the quadrupole–quadrupole interaction. For two π-orbitals with arbitrary quadrupole moments 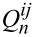 and 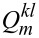 and centers separated by a displacement 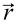, the expressions for these are

[22]
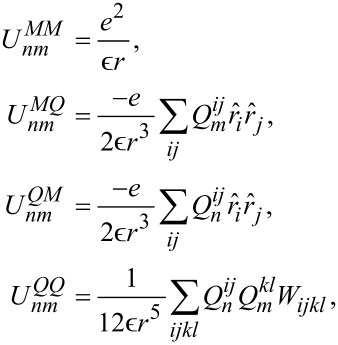


where

[23]
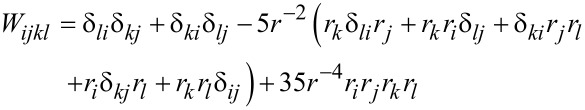


is a rank-4 tensor that characterizes the interaction of two quadrupoles and 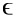 is a dielectric constant included to account for the polarizability of the core and σ electrons. Here *i, j, k*, and *l* are the Cartesian indices of the foregoing tensors and vectors. Altogether, this provides an expression for the interaction energy that is correct up to fifth order in the interatomic distance.

#### Benzene

The adjustable parameters in our Hamiltonian for gas-phase benzene are the nearest-neighbor tight-binding matrix element *t*, the on-site repulsion *U*, the dielectric constant 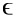, and the π-orbital quadrupole moment *Q*. These were renormalized by fitting to experimental values that should be accurately reproduced within a π-electron only model. In particular, we simultaneously optimized the theoretical predictions of (1) the six lowest singlet and triplet excitations of the neutral molecule, (2) the vertical ionization energy, and (3) the vertical electron affinity. The optimal parametrization for the π-EFT was found to be *t* = 2.70 eV, *U* = 9.69 eV, *Q* = −0.65 *e*Å^2^ and 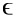 = 1.56 with a RMS relative error of 4.2 percent in the fit of the excitation spectrum. It would be interesting to compare the values of *U* and *Q* determined by this analysis with estimates from ab initio methods such as density functional theory. Note, however, that the use of “improved” values of the parameters in our effective Hamiltonian is unlikely to improve agreement with the experimental data that we considered, precisely because we optimized the π-EFT predictions for these quantities.

**Figure 1:**
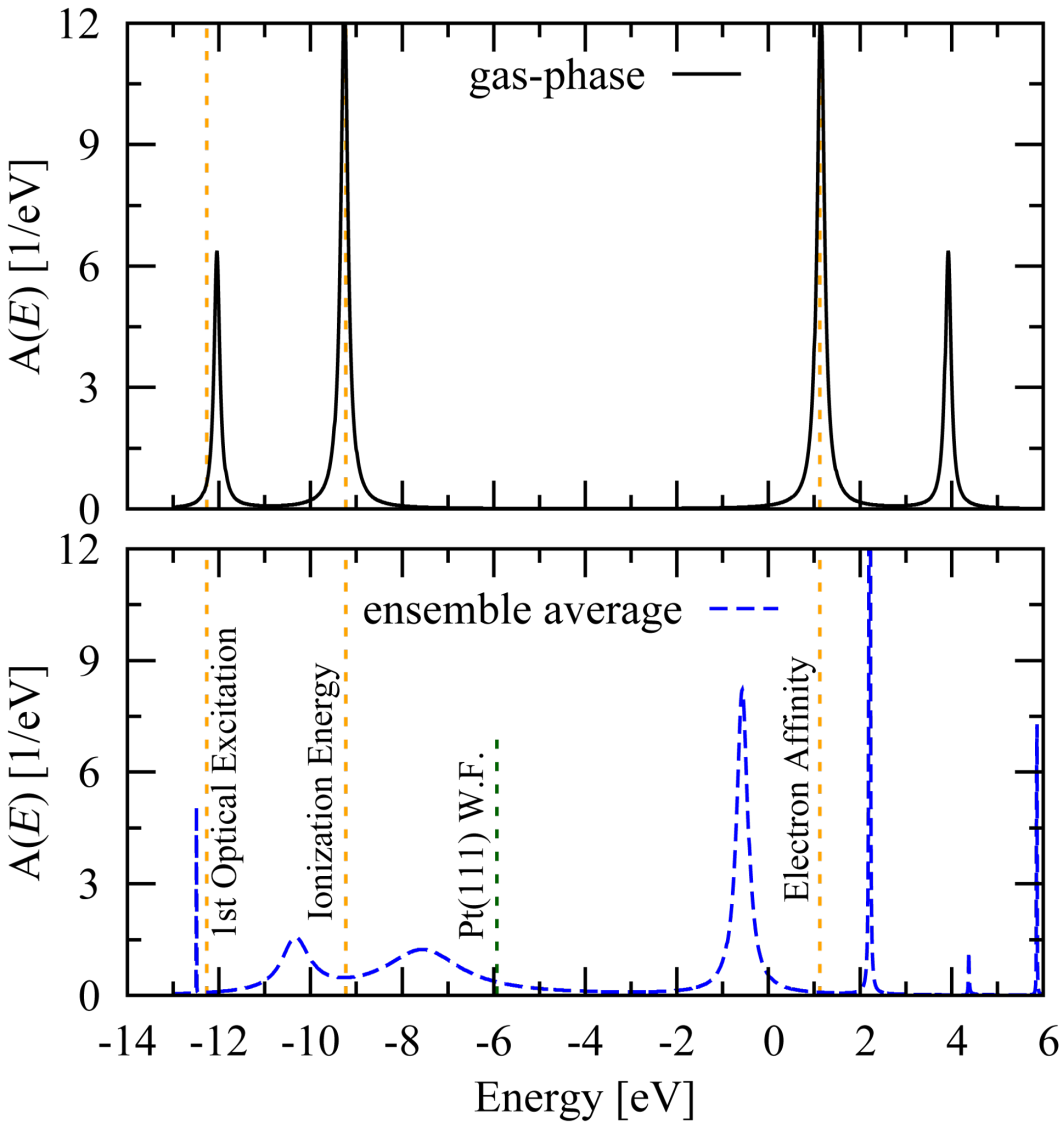
Spectral functions *A*(*E*) = −(1/π)Tr{*G*(*E*)} at room temperature for gas-phase benzene (top panel) and Pt–benzene–Pt junctions (ensemble average, bottom panel). The gas-phase resonances are broadened artificially as a guide for the eye. The dashed orange lines are fixed by (left to right) the lowest-lying optical excitation of the molecular cation [17–21], the vertical ionization energy of the neutral molecule [17–20,22], and the vertical electron affinity of the neutral molecule [23]. The asymmetry in the average spectral function arises because the HOMO resonance couples more strongly on average to the Pt tip atoms than does the LUMO resonance. The work function of the Pt(111) surface (−5.93 eV [24]) is shown for reference.

The top panel of Figure 1 shows the spectral function for gas-phase benzene within π-EFT, along with experimental values for the first optical excitation of the cation (3.04 eV), the vertical ionization energy (9.23 eV), and the vertical electron affinity (−1.12 eV). As a guide for the eye, the spectrum has been broadened artificially by using a tunneling-width matrix of Γ*_nm_* = (0.2 eV)δ*_nm_*. The close agreement between the experimental values and the maxima of the spectral function suggests that our model is accurate at this energy scale. In particular, the accuracy of the theoretical value for the lowest optical excitation of the cation is noteworthy, as this quantity was not fit during the renormalization procedure but rather represents a prediction of π-EFT.

In order to incorporate screening by metallic electrodes into π-EFT, we utilized an image multipole method whereby the interaction between an orbital and image orbitals is included up to the quadrupole–quadrupole interaction in a screened interaction matrix 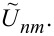 In particular, we chose a symmetric 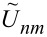 that ensures the Hamiltonian gives the energy required to assemble the charge distribution from infinity with the electrodes maintained at fixed potential, namely


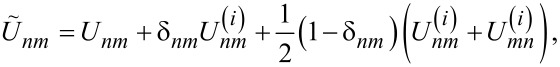


where *U_nm_* is the unscreened interaction matrix and 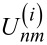 is the interaction between the *n*th orbital and the image of the *m*th orbital. When multiple electrodes are present, the image of an orbital in one electrode produces images in the others, resulting in an effect reminiscent of a hall of mirrors. We deal with this by including these “higher order” multipole moments iteratively until the difference between successive approximations of 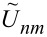 drops below a predetermined threshold.

In the particular case of the Pt–benzene–Pt junction ensemble described in the next section, the electrodes of each junction are modeled as perfect spherical conductors. An orbital with monopole moment *q* and quadrupole moment *Q^ij^* located a distance *r* from the center of an electrode with radius *R* then induces an image distribution at 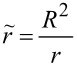 with monopole and quadrupole moments


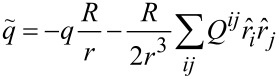


and


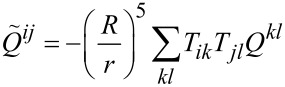


respectively. Here *T_ik_* is a transformation matrix representing a reflection about the plane that is normal to the vector 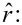



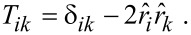


The lower panel of Figure 1 shows the Pt–benzene–Pt spectral function averaged over the ensemble of junctions described in the next section using this method. Comparing the spectrum with the gas-phase spectral function shown in the top panel of Figure 1, we see that screening due to the nearby Pt tips reduces the HOMO–LUMO gap by 33% on average, from 10.39 eV in the gas-phase to 6.86 eV over the junction ensemble.

The screening of intramolecular Coulomb interactions by nearby conductor(s) illustrated in Figure 1 leads to an attractive interaction between a molecule and a metal surface (van der Waals interaction). By diagonalizing the molecular Hamiltonian with and without the effects of screening included in *U_nm_*, it is possible to determine the van der Waals interaction at arbitrary temperature between a neutral molecule and a metallic electrode by comparing the expectation values of the Hamiltonian in these two cases:


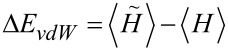


**Figure 2:**
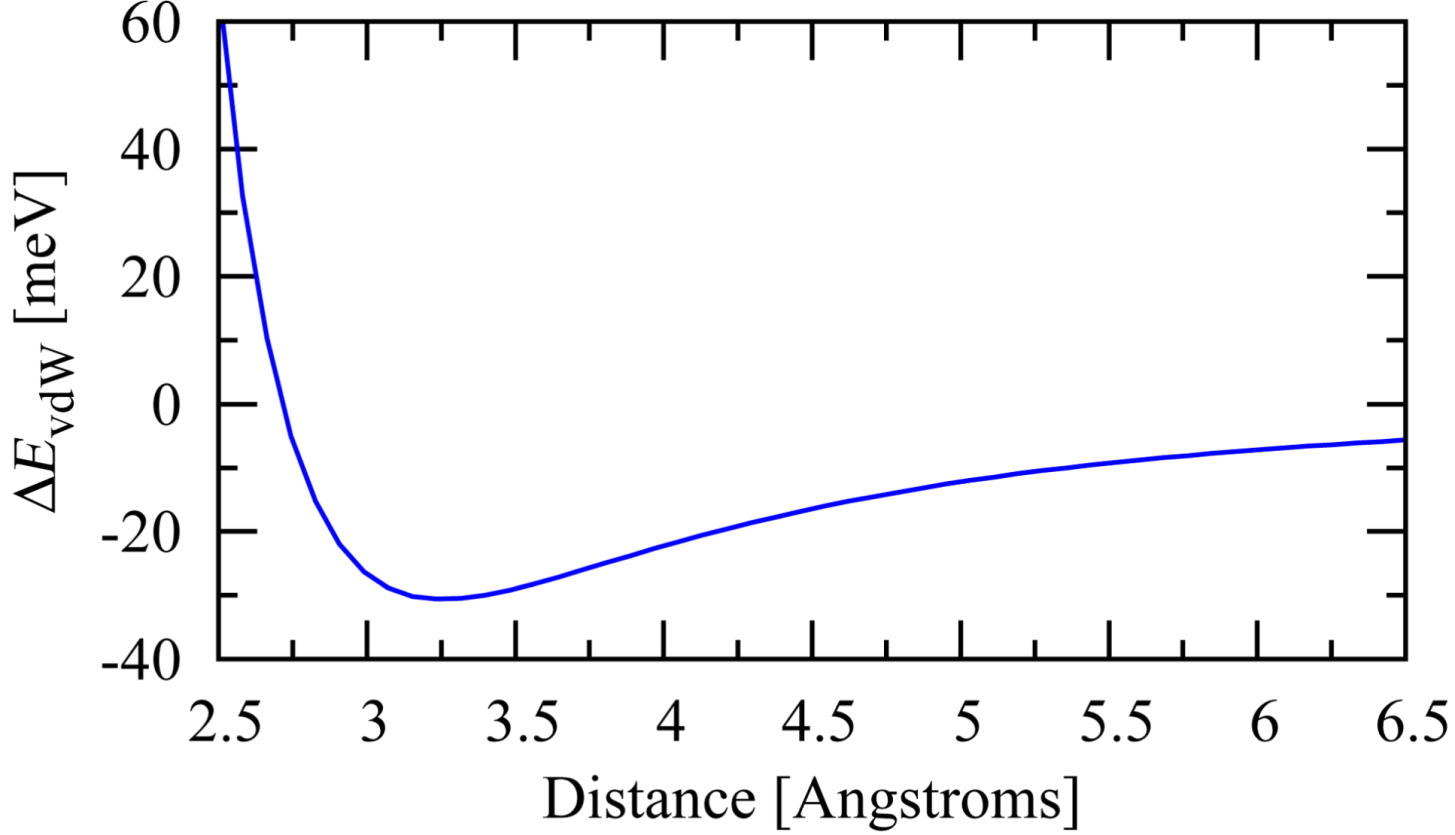
Calculated van der Waals contribution to the binding energy of benzene adsorbed on a Pt(111) surface as a function of distance. Here the plane of the molecule is oriented parallel to the Pt surface. A phenomenological short-range repulsion 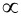 *r*^−12^ has been included to model the Pauli repulsion when the π-orbitals overlap the Pt surface states.

This procedure was carried out at zero temperature for benzene oriented parallel to the surface of a planar Pt electrode at a variety of distances, and the results are shown in Figure 2. Note that an additional phenomenological short-range repulsion proportional to *r*^−12^ has been included in the calculation to model the Pauli repulsion arising when the benzene π-orbitals overlap the Pt surface states.

### The lead–molecule coupling

When an isolated molecule is connected to electrodes and a molecular junction is formed, the energy levels of the molecule are broadened and shifted as a result of the formation of a lead–molecule bond and due to the electrostatic influence of the leads. The bonding between lead *α* and the molecule is described by the tunneling width matrix Γ*^α^* and the electrostatics, including intramolecular screening and van der Waals effects, are described by the effective molecular Hamiltonian derived using the aforementioned π-EFT. Although we use the Pt–benzene–Pt junction as an example here, the techniques we discuss are applicable to any conjugated organic molecular junction.

#### Bonding

The bonding between the tip of electrode *α* and the molecule is characterized by the tunneling-width matrix Γ*^α^* given by Equation 4. When a highly conductive SMJ [4] is formed, the lead and molecule are in direct contact such that the overlap between the π-electron system of the molecule and *all* of the quasi-atomic wavefunctions of the atomically sharp electrode are relevant. In this case we may express the elements of Γ*^α^* as [6]

[24]
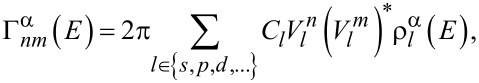


where the sum is calculated over the evanescent tunneling modes emanating from the metal tip, labeled by their angular-momentum quantum numbers, 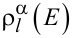 is the local density of states on the apex atom of electrode *α*, and 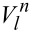 is the tunneling matrix element of orbitals *l* [25]. The constants *C_l_* can in principle be determined by matching the evanescent tip modes to the wavefunctions within the metal tip [25]; however, we set 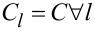 and determine the constant *C* by fitting to the peak of the experimental conductance histogram [4]. In the calculation of the matrix elements, we use the effective Bohr radius of a π-orbital a^*^ = *a*_0_/*Z*, where *a*_0_ ≈ 0.53 Å is the Bohr radius and *Z* = 3.22 is the effective hydrogenic charge associated with the π-orbital quadrupole moment −0.65 *e*Å^2^, determined by π-EFT.

For each Pt tip, we include one *s*, three *p* and five *d* orbitals in our calculations, which represent the evanescent tunneling modes in free space outside the apex atom of the tip. At room temperature, the Pt density of states (DOS) ρ*^α^*(*E*) = Σ*_l_*ρ*_l_^α^*(*E*) is sharply peaked around the Fermi energy [26] with ρ*^α^*(ε*_F_*) = 2.88/eV [27]. In accordance with [25], we distribute the total DOS such that the *s* orbital contributes 10%, the *p* orbitals contribute 10%, and the *d* orbitals contribute 80%.

**Figure 3:**
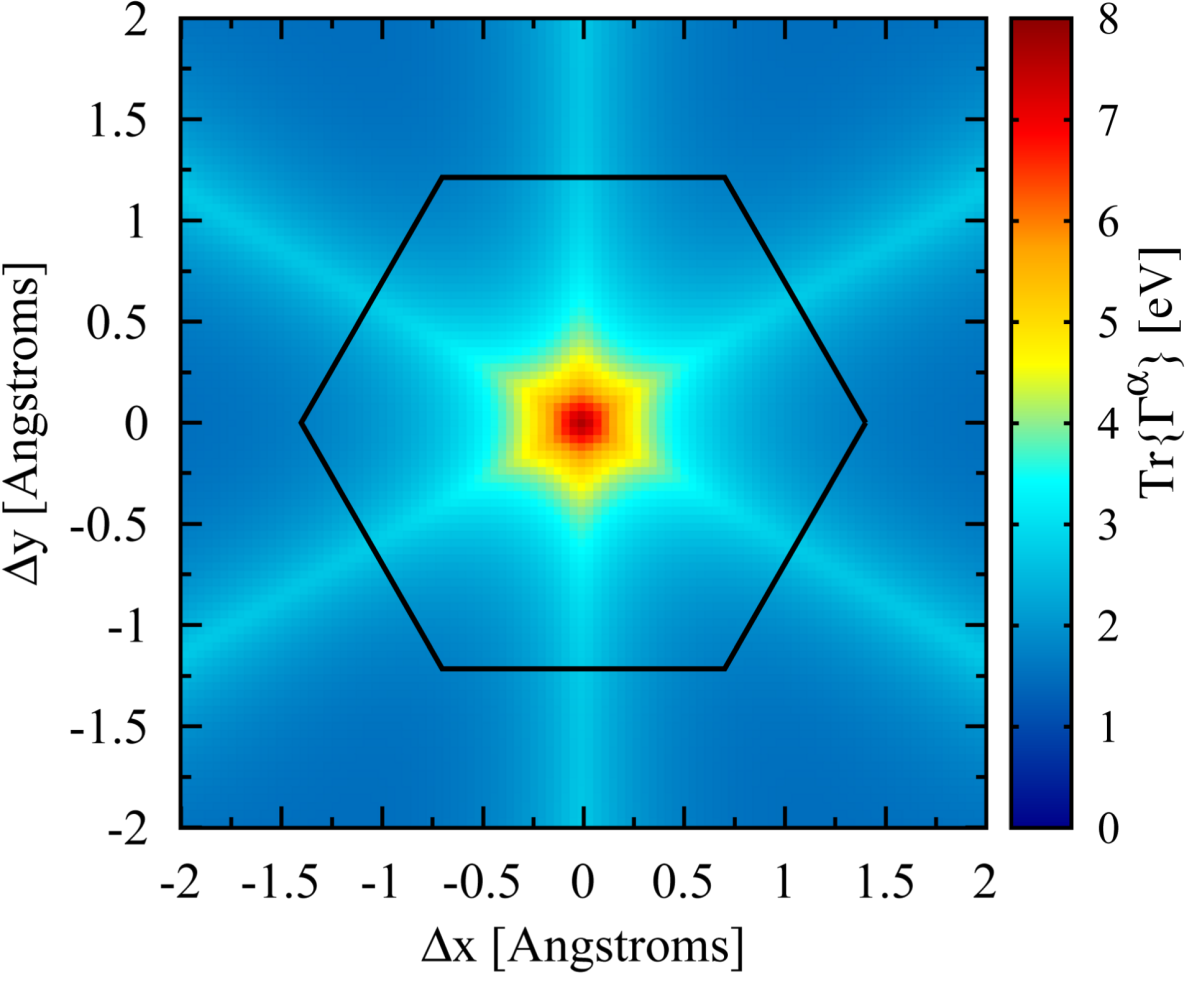
The trace of Γ*^α^* for a Pt electrode in contact with a benzene molecule. Nine total basis states of the Pt tip are included in this calculation (one *s*, three *p* and five *d* states). The tip height above the plane of the molecule is adjusted at each point such that the Pt–C distance is fixed to 2.65 Å (see text). Tr{Γ*^α^*} retains the (six-fold) symmetry of the molecule and is sharply peaked near the center of the benzene ring, indicating that the strongest bonds are formed when the lead is in the “atop” configuration. The benzene molecule is shown schematically with the black lines; the carbons atoms are located at each vertex.

We are interested in investigating transport through stable junctions where the “atop” binding configuration of benzene on Pt has the largest binding energy [28–30]. In this configuration, the distance between the tip atom and the center of the benzene ring is ≈2.25 Å [4], giving a tip to orbital distance of ≈2.65 Å (the C–C bond distance is taken as 1.4 Å). The trace of Γ*^α^*(ε*_F_*) is shown as a function of tip position in Figure 3, where for each tip position the height was adjusted such that the distance to the closest carbon atom was 2.65 Å. From the figure, it is evident that the lead–molecule coupling strength is peaked when the tip is in the vicinity of the center of the benzene ring (whose outline is drawn schematically in black). As shown in [2], the hybridization contribution to the binding energy is


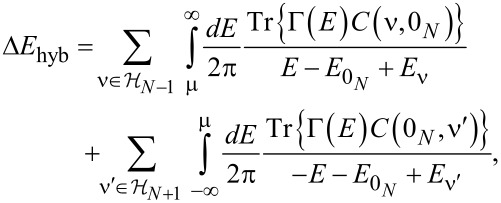


which is roughly 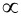
Tr{Γ(ε*_F_*)}. Here μ is the chemical potential of the lead metal, 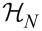 is the *N*-particle molecular Hilbert space, and 0*_N_* is the ground state of the *N*-particle manifold of the neutral molecule. The sharply peaked nature of Tr{Γ*^α^*} seen in Figure 3 is thus consistent with the large binding energy of the atop configuration.

This result motivates our procedure for generating the ensemble of junctions, in which we consider the tip position in the plane parallel to the benzene ring to be a 2-D Gaussian random variable with a standard deviation of 0.25 Å, chosen to correspond with the preferred bonding observed in this region. For each position, the height of each electrode (one placed above the plane and one below) is adjusted such that the closest carbon to the apex atom of each electrode is at a distance of 2.65 Å. Each lead is positioned independently of the other. This procedure ensures that the full range of possible, bonded junctions are included in the ensemble.

**Figure 4:**
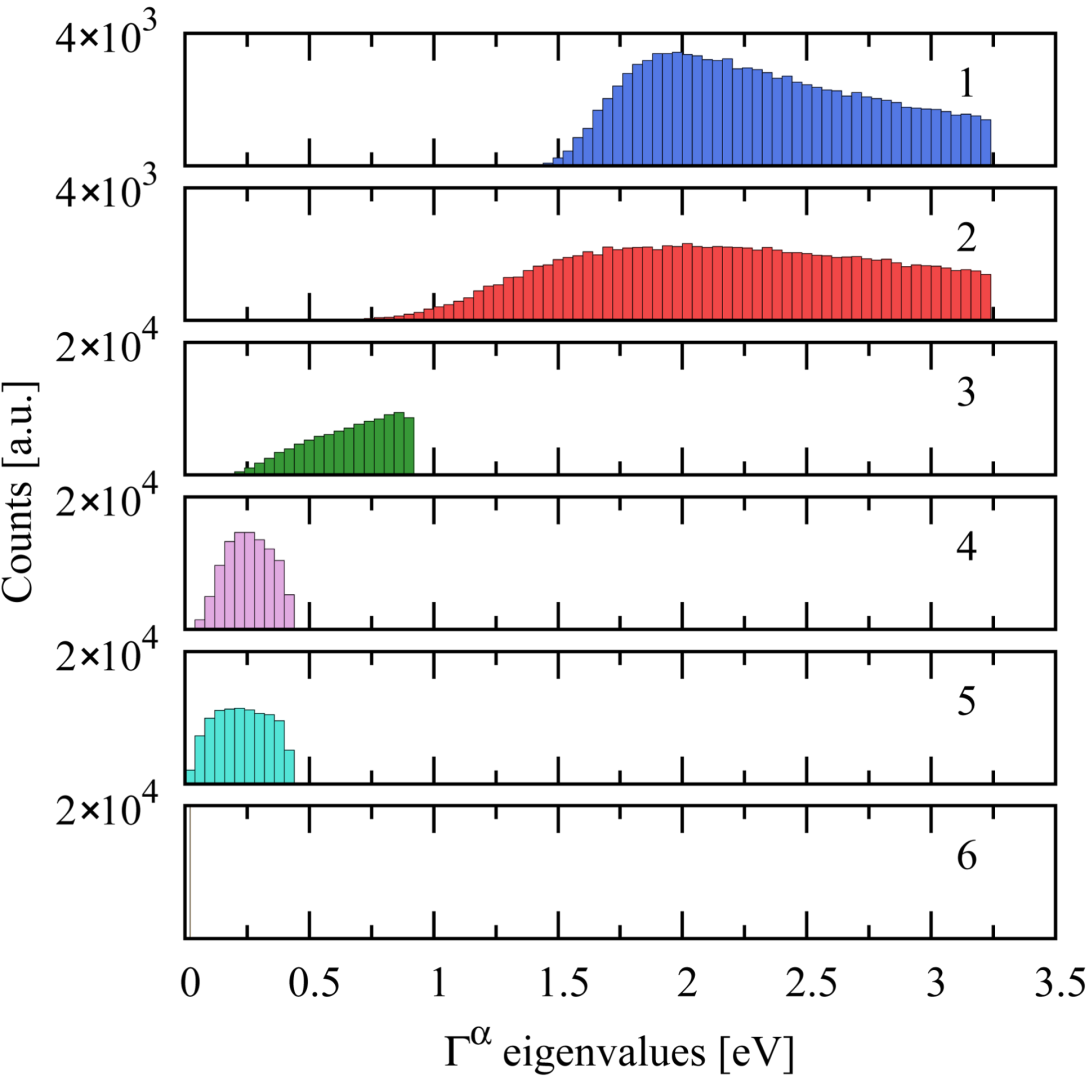
Eigenvalue decomposition of an ensemble of Γ*^α^* matricies, showing that each lead–molecule contact has ~5 channels. Note that nine orthogonal basis orbitals were included in the calculation for each lead.

**Figure 5:**
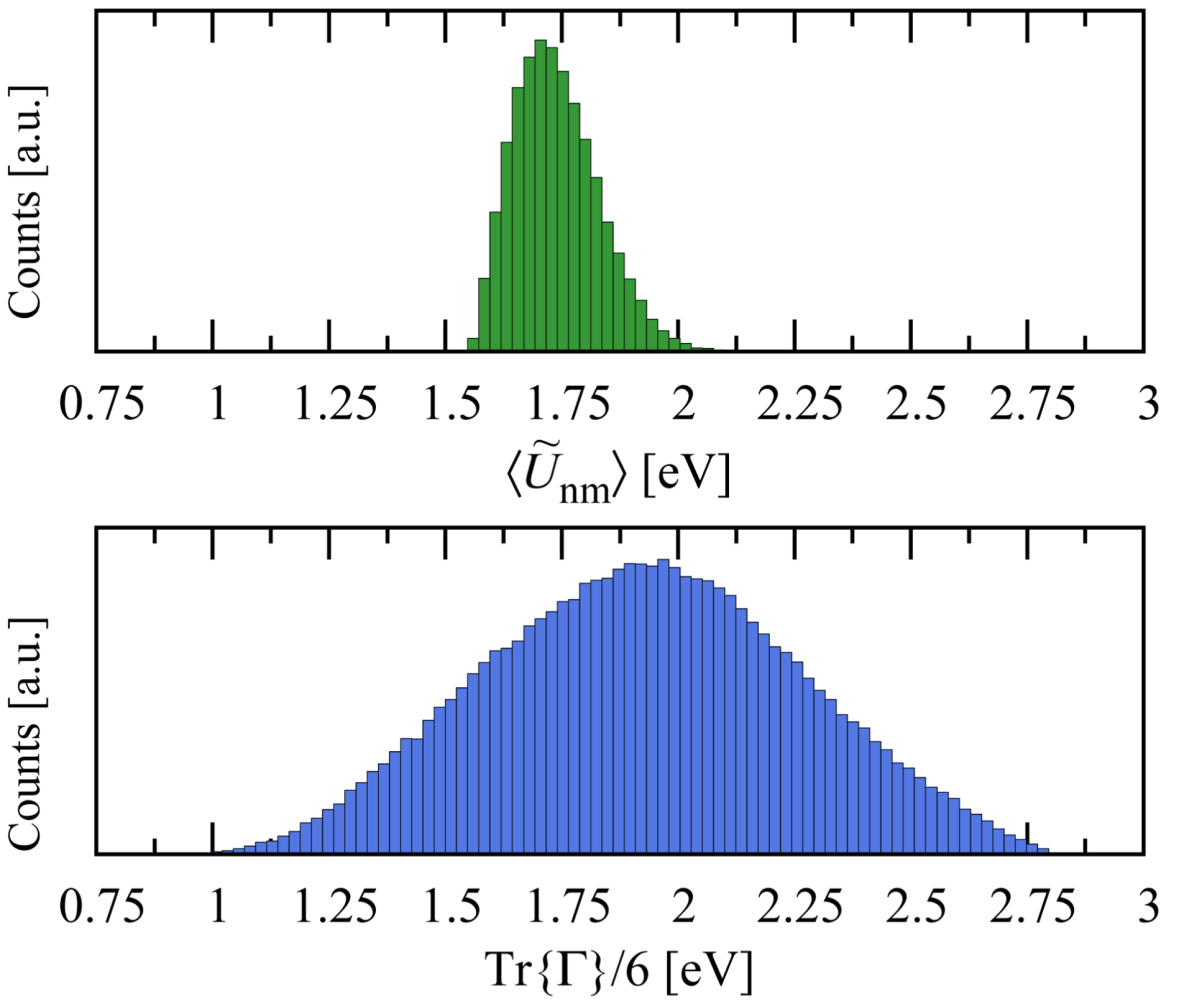
The distribution of charging energy 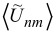 (top panel) and Tr{Γ} (bottom panel) over the ensemble described in the text. Here Γ = Γ^1^ + Γ^2^ is the total tunneling-width matrix of the junction. The width of the Tr{Γ} distribution is about four times that of the 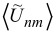 distribution. The peaks of the 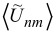 and Tr{Γ}/6 distributions are at 1.68 eV and 1.95 eV, respectively, suggesting that transport occurs in an intermediate regime in which both the particle-like and wave-like character of the charge carriers must be considered.

The eigenvalue distributions of Γ*^α^* over the ensemble are shown in Figure 4. Although we include nine (orthogonal) basis orbitals for each lead, the Γ matrix only exhibits five nonzero eigenvalues, presumably because only five linear combinations can be formed that are directed toward the molecule. Although we have shown the distribution for a single lead, the number of transmission channels for two leads, where each Γ*^α^* matrix has the same rank, will be the same even though the overall lead–molecule coupling strength will be larger. The average coupling per orbital with two electrodes is shown in the bottom panel of Figure 5.

#### Screening

The ensemble of screened interaction matrices 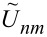 is generated using the same procedure discussed above. Each Pt electrode is modelled as a conducting sphere with radius equal to the Pt polarization radius (1.87 Å). This is equivalent to the assumption that screening is due mainly to the apex atoms of each Pt tip. The screening surface is placed such that it lies one covalent radius away from the nearest carbon atom [14].

The average over the interaction matrix elements 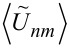 defines the “charging energy” of the molecule in the junction [14]. The charging energy 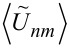 and per-orbital Tr{Γ} distributions are shown in the top and bottom panels of Figure 5, respectively, in which two electrodes are used in all calculations. As indicated by the figure, the Tr{Γ}/6 distribution is roughly four times as broad as the charging-energy distribution. This fact justifies the use of the ensemble-average 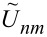 matrix for transport calculations [2], an approximation which makes the calculation of thousands of junctions computationally tractable. The peak values of the 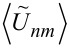 and Tr{Γ}/6 distributions are 1.68 eV and 1.95 eV, respectively, suggesting that transport occurs in an intermediate regime in which both the particle-like and wave-like character of the charge carriers must be considered.

In addition to sampling various bonding configurations, we also consider an ensemble of junctions to sample all possible Pt surfaces. The work function of Pt ranges from 5.93 eV for the (111) surface to 5.12 eV for the (331) surface [24], and we assume that μ_Pt_ is distributed uniformly over this interval.

**Figure 6:**
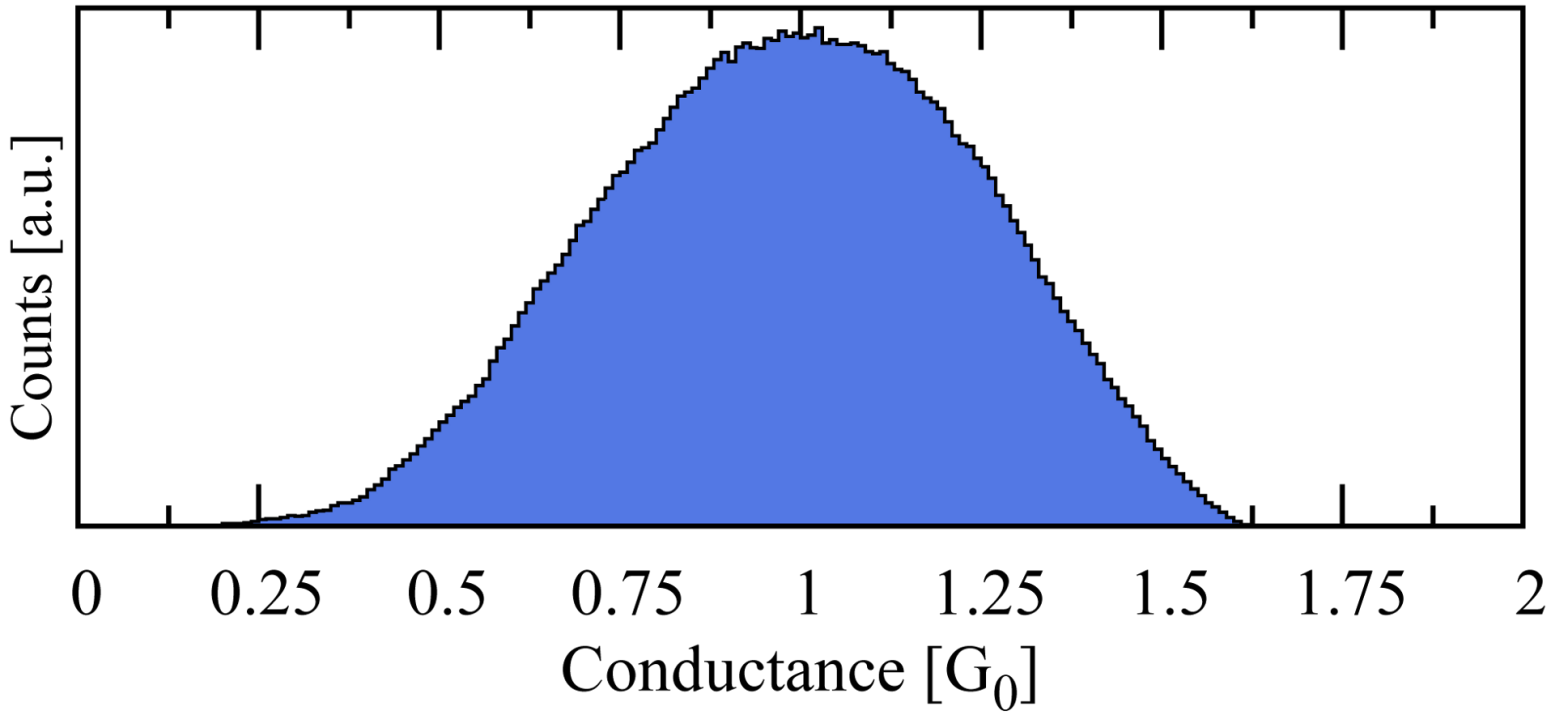
Calculated conductance histogram for the ensemble over bonding configurations and Pt surfaces. The value of the conductance peak has been fit to match the experimental data [4], determining the constant *C* in Equation 24. There is no peak for *G* ~ 0 because we designed an ensemble of junctions where both electrodes are strongly bound to the molecule.

Using this ensemble, the conductance histogram over the ensemble of junctions can be computed, and is shown in Figure 6. The constant prefactor *C* appearing in the tunneling matrix elements [25] in Equation 24 was determined by fitting the peak of the calculated conductance distribution to that of the experimental conductance histogram [4]. Note that the width of the calculated conductance peak is also comparable to that of the experimental peak [4].

## Results and Discussion

**Figure 7:**
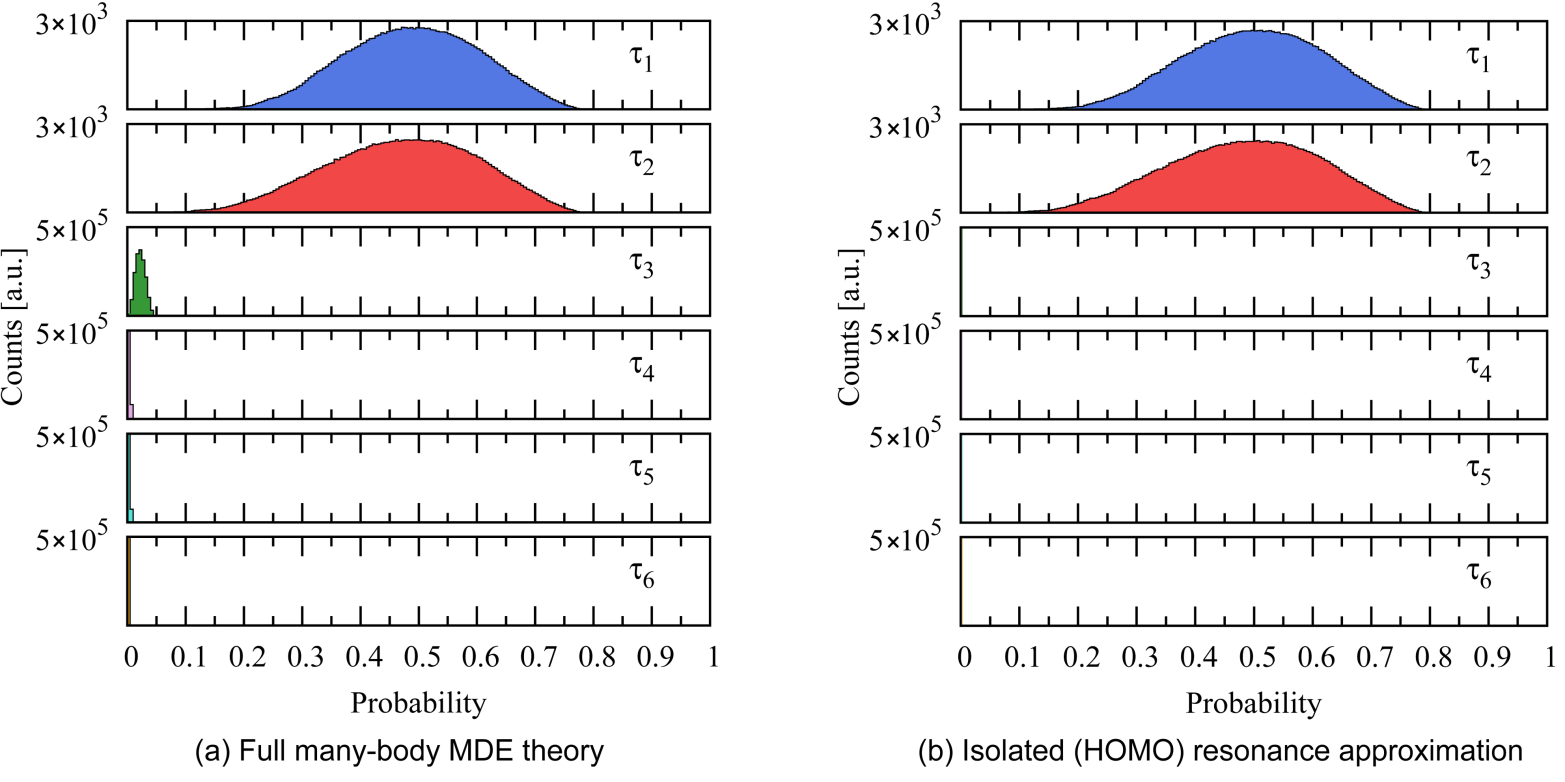
The calculated eigenvalue distributions for an ensemble of 1.74 × 10^5^ (2000 bonding configurations × 87 Pt surfaces) Pt–benzene–Pt junctions using many-body theory with (a) the full spectrum and (b) the isolated-resonance approximation for the (doubly degenerate) HOMO resonance. Despite each lead forming ~5 bonds (cf. Figure 4), calculations in both cases exhibit only two dominant channels, which arise from the degeneracy of the relevant (HOMO) resonance. The weak third channel seen in (a) is a consequence of the large lead–molecule coupling and is consistent with the measurements of [4].

The transmission eigenvalue distributions for ensembles of 1.74 × 10^5^ Pt–benzene–Pt junctions calculated by using the full many-body spectrum and in the isolated-resonance approximation are shown in Figure 7a and Figure 7b, respectively. Despite the existence of five covalent bonds between the molecule and each lead (cf. Figure 4), there are only two dominant transmission channels, which arise from the two-fold-degenerate HOMO resonance closest to the Pt Fermi level [2]. As proof of this point, we calculated the transmission eigenvalue distribution, over the same ensemble, using only the HOMO resonance in the isolated-resonance approximation (Equation 19). The resulting transmission eigenvalue distributions, shown in Figure 7b, are nearly identical to the full distribution shown in Figure 7a, with the exception of the small but experimentally resolvable [4] third transmission channel.

The lack of a third channel in the isolated-resonance approximation is a direct consequence of the two-fold degeneracy of the HOMO resonance, which can therefore contribute at most two transmission channels. The third channel thus arises from further off-resonant tunneling. In fact, we would argue that the very observation of a third channel in some Pt–benzene–Pt junctions [4] is a consequence of the very large lead–molecule coupling (~2 eV per atomic orbital) in this system. Having simulated junctions with electrodes whose DOS at the Fermi level is smaller than that of Pt, we expect junctions with Cu or Au electrodes, for example, to exhibit only two measurable transmission channels.

**Figure 8:**
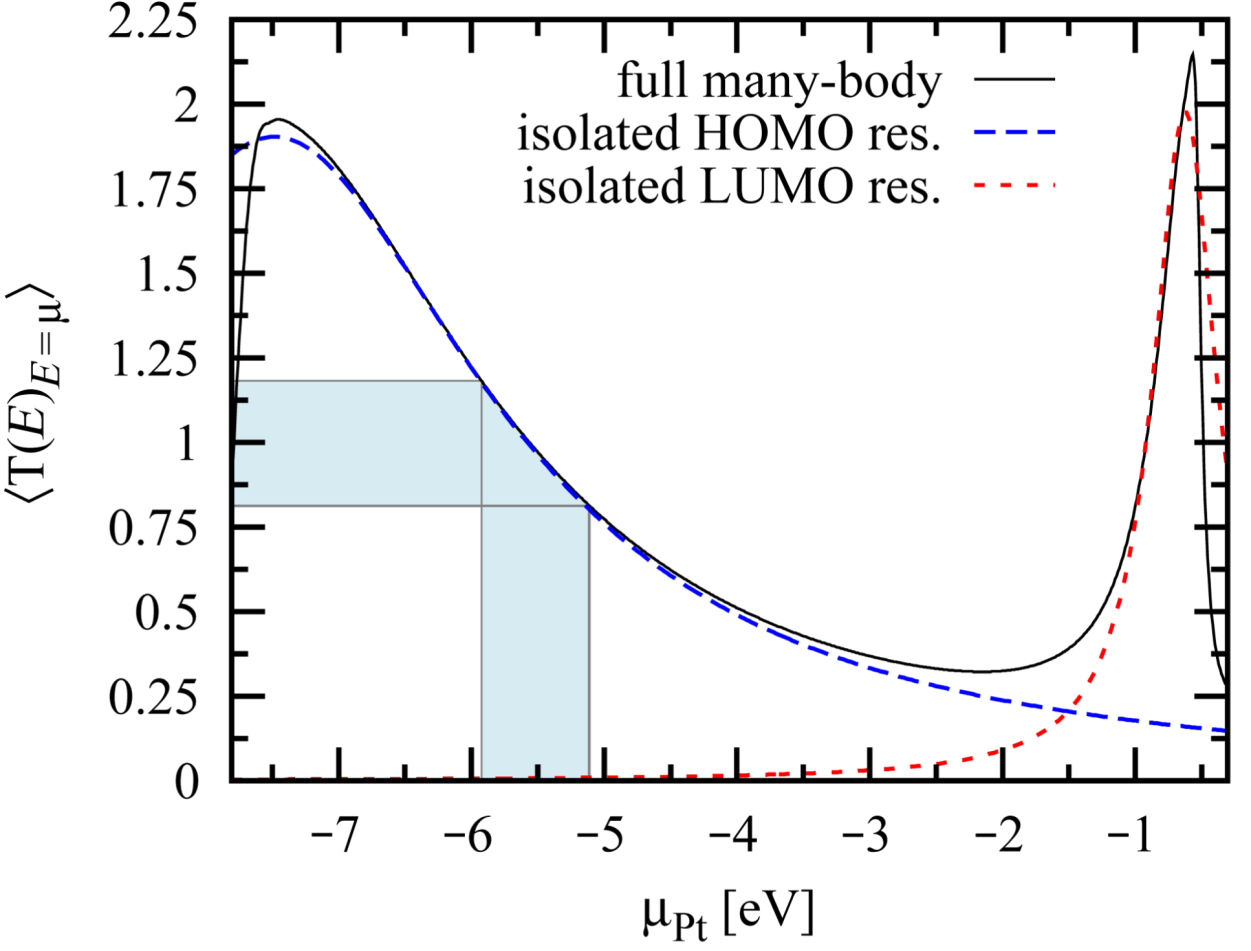
The calculated average total transmission averaged over 2000 bonding configurations through a Pt–benzene–Pt junction shown as a function of the chemical potential of the leads μ_Pt_. The isolated-resonance approximation employing the HOMO or LUMO resonance accurately describes the full many-body transport in the vicinity of the HOMO or LUMO resonance, respectively. These data are in good agreement with the measurements of [4]. The work-function range for the crystal planes of Pt is shaded in blue, where −5.93 eV ≤ μ_Pt_ ≤ −5.12 eV [24].

In order to investigate the efficacy of the isolated-resonance approximation further, we calculated the average total transmission through a Pt–benzene–Pt junction. The transmission spectra calculated using the full molecular spectrum, the isolated HOMO resonance and the isolated LUMO resonance are each shown as a function of the chemical potential of the leads μ_Pt_ in Figure 8. The spectra are averaged over 2000 bonding configurations and the blue shaded area indicates the range of possible chemical potentials for the Pt electrodes. The close correspondence between the full transmission spectrum and the isolated HOMO resonance over this range is consistent with the accuracy of the approximate method shown in Figure 7. Similarly, in the vicinity of the LUMO resonance, the isolated LUMO resonance approximation accurately characterizes the average transmission. The HOMO–LUMO asymmetry in the average transmission function arises because the HOMO resonance couples more strongly on average to the Pt tip atoms than does the LUMO resonance.

It is tempting to assume, based on the accuracy of the isolated-resonance approximation in our many-body transport theory, that an analogous “single molecular orbital” approximation would also be sufficient in a transport calculation based, e.g., on density-functional theory (DFT). However, this is not the case. Although the isolated-resonance approximation can also be derived within DFT, in practice, it is necessary to use an “extended molecule” to account for charge transfer between molecule and electrodes. Analyzing transport in terms of extended molecular orbitals has proven problematic. For example, the resonances of the extended molecule in [31] apparently accounted for less than 9% of the current through the junction.

Employing an extended molecule also makes it difficult, if not impossible, to interpret transport contributions in terms of the resonances of the molecule itself [31]. Since charging effects in SMJs are well-described in our many-body theory [6,12], there is no need to utilize an extended molecule, and therefore the resonances in our isolated-resonance approximation are true molecular resonances.

The full counting statistics of a distribution are characterized by its cumulants. By using a single-particle theory to describe a single-channel junction, it can be shown [32–33] that the first cumulant is related to the junction transmission function, while the second cumulant is related to the shot-noise suppression. Often this suppression is phrased in terms of the Fano factor [34]

[25]
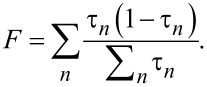


**Figure 9:**
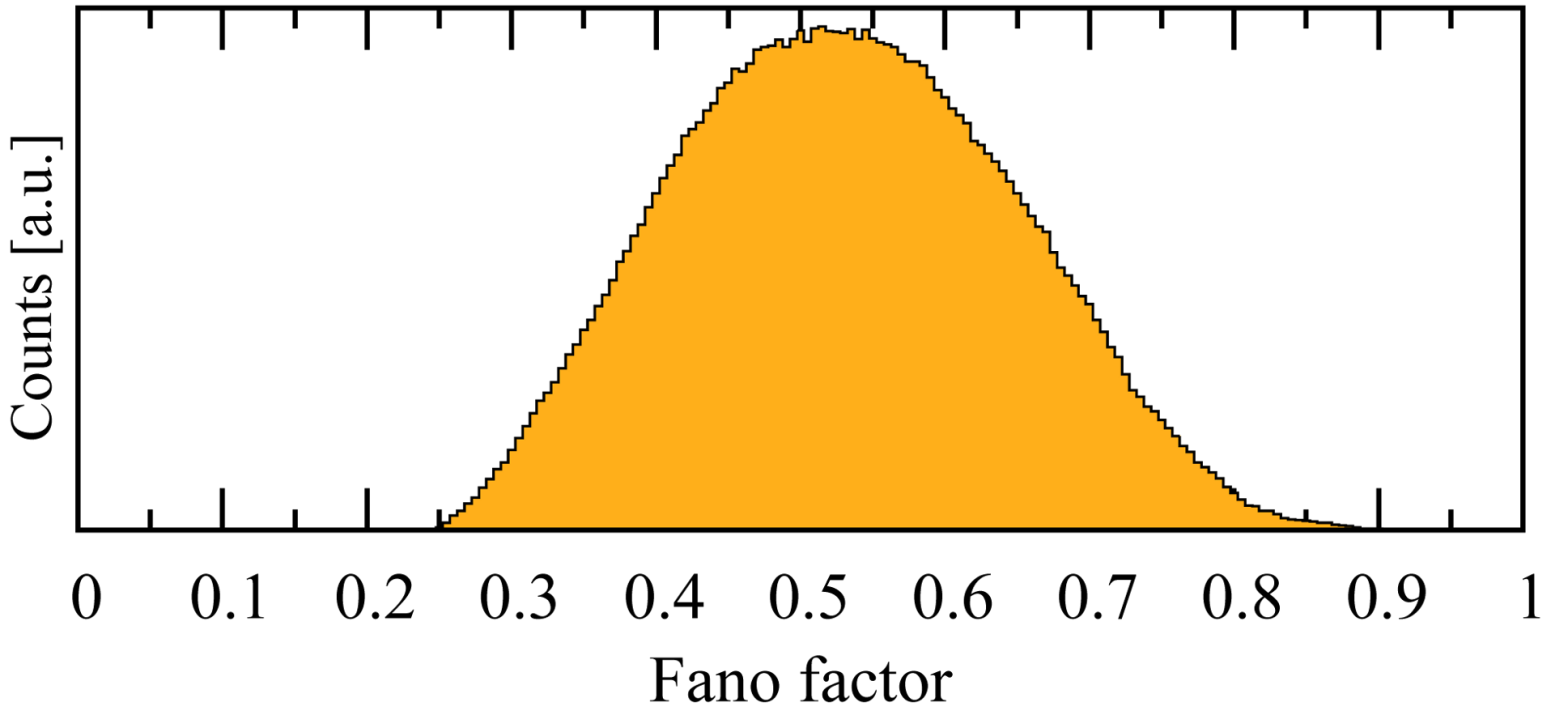
The calculated Fano factor *F* distribution for the full ensemble of 1.74 × 10^5^ Pt–benzene–Pt junctions. *F* describes the nature of the transport, where *F* = 0 and *F* = 1 characterize wave-like and particle-like transport, respectively. The peak value of this distribution *F* ~ 0.51 indicates that we are in an intermediate regime.

In Figure 9 we show the distribution of *F* for our ensemble of junctions, where the τ*_n_* have been calculated from many-body theory. Because of the fermionic character of the charge carriers, 0 ≤ *F* ≤ 1 , with *F* = 0 corresponding to completely wavelike transport, and a value of *F* = 1 corresponding to completely particle-like transport. From the figure, we see that *F* is peaked at ~0.51 implying that both the particle *and* wave aspects of the carriers are important, a fact which is consistent with the commensurate charging energy and bonding strength (cf. Figure 5).

In such an intermediate regime both “complementary” aspects of the charge carriers are equally important, requiring a many-body description and resulting in many subtle and interesting effects. For example, the transport in this regime displays a variety of features stemming from the interplay between Coulomb blockade and coherent-interference effects, which occur simultaneously [6,11]. Although the Fano factor reflects the nature of the transport, it is not directly related to the shot-noise power in a many-body theory. The richness of the transport in this regime, however, suggests that a full many-body calculation of a higher-order moment, such as the shot noise, may exhibit equally interesting phenomena.

## Conclusion

We have developed a state-of-the-art technique to model the lead–molecule coupling in highly conductive molecular junctions. The bonding between the lead and molecule was described by using an “ab initio” model in which the tunneling matrix elements between all relevant lead tip wavefunctions and the molecule were included, producing multi-channel junctions naturally from a physically motivated ensemble over various contact geometries. Coulomb interactions between the molecule and the metallic leads were included by using an image multipole method within π-EFT. In concert, these techniques allowed us to accurately model SMJs within our many-body theory.

The transport for an ensemble of Pt–benzene–Pt junctions, calculated by using our many-body theory, confirmed our previous finding [2] that the number of dominant transmission channels is two, with the higher channels more strongly suppressed within the more realistic treatment of lead-molecule coupling presented here. Moreover, we find that the transport through a Pt–benzene–Pt junction can be accurately described by using only the relevant (two-fold-degenerate HOMO) molecular resonance. The exceptional accuracy of such an isolated-resonance approximation, however, may be limited to small molecules with large charging energies. In larger molecules, where the charging energy is smaller, further off-resonant transmission channels may become more important.

In metallic point contacts the number of channels is completely determined by the valence of the metal. Despite the larger number of states available for tunneling transport in SMJs, we predict that the number of transmission channels is typically more limited than in single-atom contacts because molecules are less symmetric than atoms. Channel-resolved transport measurements of SMJs therefore offer a unique probe into the symmetry of the molecular species involved.

## Supporting Information

We investigate the origin of the transmission distribution widths by considering transport ensembles over Pt surfaces with fixed bonding, and over bonding configurations with a fixed Pt surface.

File 1 Transport distribution width decomposition
