# Supplementary material for: Transmission eigenvalue distributions in highly conductive molecular junctions
Source: Beilstein J Nanotechnol. 2012 Jan 16;3:40–51. doi: 10.3762/bjnano.3.5 (PMC3304317; doi:10.3762/bjnano.3.5)
Supplement: File 1 — Transport distribution width decomposition [file Beilstein_J_Nanotechnol-03-40-s001.pdf]

Supporting Information

for

Transmission eigenvalue distributions in highly conductive molecular junctions

Justin P. Bergfield*[1], Joshua D. Barr[2] and Charles A. Stafford[2]

Address: [1]Departments of Chemistry and Physics, University of California, Irvine, California 92697, USA and [2]Department of Physics, University of Arizona, 1118 East Fourth Street, Tucson, AZ 85721

Email: Justin P. Bergfield - jbergfie@uci.edu

* Corresponding author

Transport distribution width decomposition

Here we investigate the origin of the width of the Pt–benzene–Pt transport distributions by considering transport ensembles generated (a) by using a fixed-bonding configuration but varying the Pt surfaces only, or (b) with a fixed Pt surface but varying the bonding configurations only. In the first ensemble, each lead was assigned the average bonding configuration, found by averaging Γ^α over the ensemble, and the ensemble was constructed by varying over all Pt crystal planes, where -5.84 eV $\leq \mu_{\mathrm{Pt}} \leq 5.12$ eV [1]. In the second ensemble the average Pt surface ($\mu_{\mathrm{Pt}} = -5.48$ eV) was chosen from the work function range and the ensemble was generated by varying the bonding configurations as described in the main text.

The calculated transmission eigenvalue distribution for an ensemble of Pt–benzene–Pt junctions, found by varying only the Pt surface, is shown in Figure 1a. Γ^α for each lead is given by $\langle\Gamma^\alpha\rangle$, which is the tunneling-width matrix averaged over the 2000 bonding configurations used in the

main article. As indicated in the figure, two dominant channels and one weak channel are observed. The first two channels exhibit a nearly uniform distribution over the interval 0.4 to 0.6, owing to the nearly linear dispersion of the transmission function over the Pt work-function range (cf. Figure 8 in the main text).

The calculated transmission eigenvalue distribution for an ensemble of Pt–benzene–Pt over 2000 bonding configurations at fixed $\mu_{\mathrm{Pt}} = -5.48$ eV is shown in Figure 1b. The full-width at half maximum of each of the distributions of the first two channels is ~ 0.3, which is significantly broader than that for the fixed-bonding-configuration ensemble shown in Figure 1a.

Finally, we calculate the conductance histograms for each ensemble method, which are shown in Figure 2, along with the histogram of both ensembles taken from the main article. Both the transmission eigenvalue and conductance distributions suggest that the width of the transport distributions are governed predominantly by the width of the bonding-configuration ensemble.

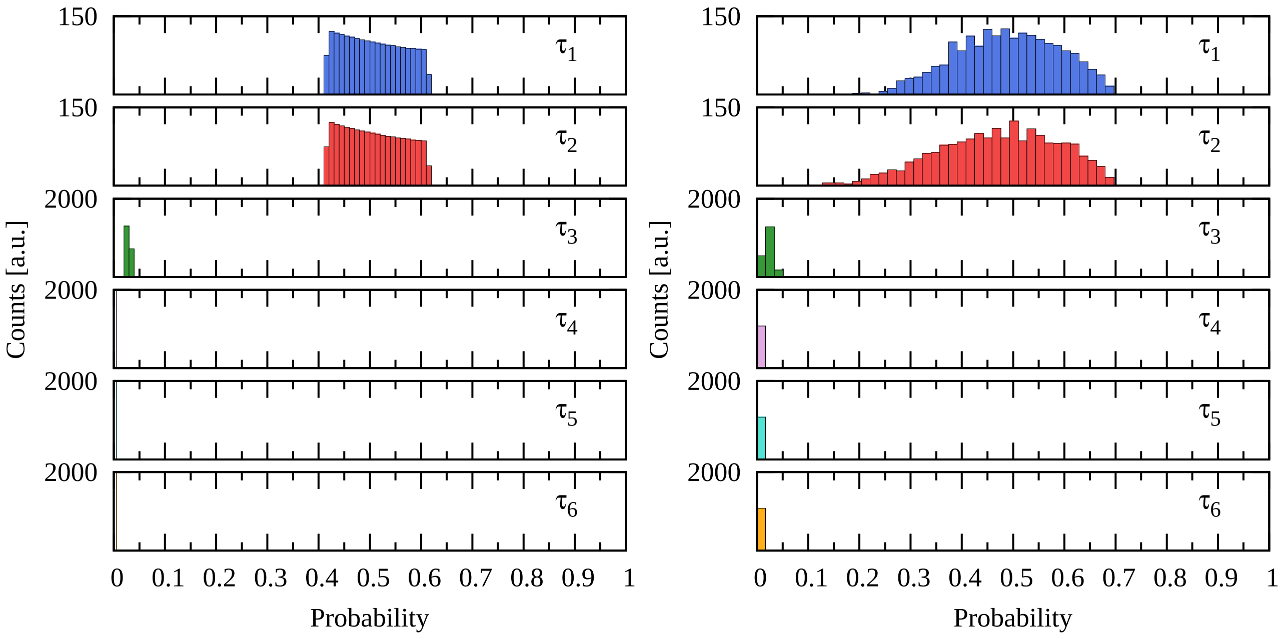

(a) Fixed Bonding Configuration Ensemble

(b) Fixed Pt Surface Ensemble

Figure 1: The calculated eigenvalue distributions for ensembles of Pt–benzene–Pt junctions using many-body theory. (a) The ensemble contains over 5000 Pt surfaces with the bonding configuration fixed to the average over the 2000 member ensemble discussed in the main text. Three channels are observed with the first and second exhibiting nearly uniform distributions over the interval 0.4 to 0.6, while the peak of the weak third channel occurs around 0.03. (b) The ensemble is over 2000 bonding configurations with $\mu_{\mathrm{Pt}} = -5.48$ eV. The full width at half-maximum of the dominant channel distributions in (b) is ~ 0.3, which roughly ~ 1.5 times that of ensemble (a) and closely resembles the distribution width of the full ensemble used in the main article.

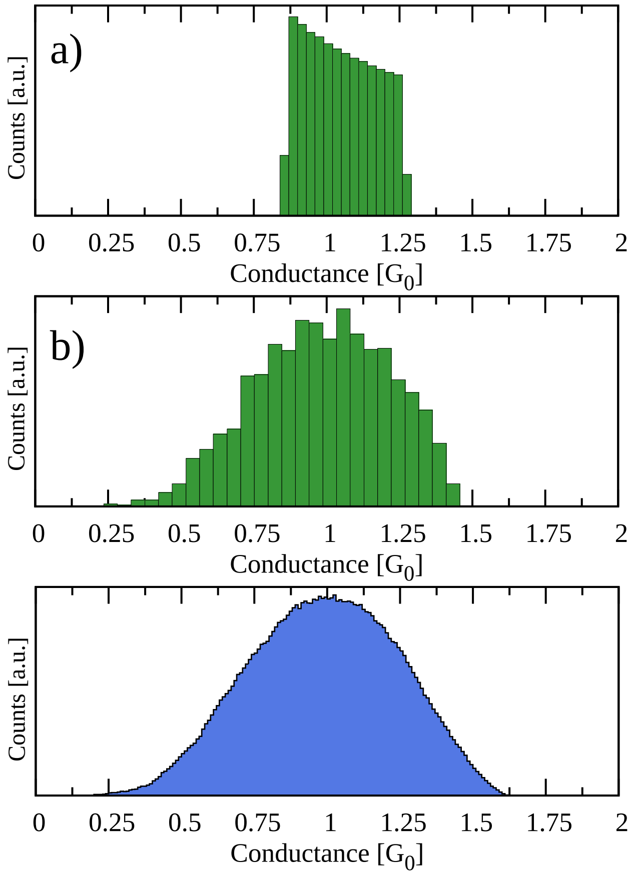

Figure 2: The calculated conductance histograms using ensembles over (a) various Pt surfaces, (b) various bonding configurations, and (bottom histogram) both. Comparing the full histogram (Figure 6 in the main article, bottom panel here) to those generated over each ensemble, it is evident that the bonding ensemble most strongly dictates the width of the conductance distribution.

References

1. *CRC Handbook of Chemistry and Physics*; Lide, D. R., Ed.; CRC Press: Boca Raton, FL, 2005.
